# The Limbic Brain, Moral Distress and Ethical Decision‐Making in Nursing Practice

**DOI:** 10.1111/nup.70087

**Published:** 2026-04-22

**Authors:** Alicia Wickens

**Affiliations:** ^1^ Athabasca University Athabasca Canada

**Keywords:** affective neuroscience, clinical judgement, ethical decision‐making, limbic brain, moral distress, moral resilience, nursing ethics

## Abstract

Nursing practice unfolds at the intersection of the body's regulatory systems and the emotional and social realities of care. The paper examines how the limbic brain mediates between the body's internal environment, interoception, allostatic regulation and autonomic arousal, and the external social world shaped by hierarchy, norms, power and institutional pressures. It also explores how this mediating role influences moral perception and clinical decision‐making. Drawing on Damasio's somatic marker hypothesis and contemporary work on affective neuroscience, I analyse the strengths and limitations of the claim that emotion is not opposed to reason but an essential element of it. I integrate the complementary neurocognitive perspectives of predictive processing, interoceptive inference and the neural architecture of moral emotion, with nursing ethics literature on moral distress, to show how affective states can both guide and distort clinical judgement, particularly under chronic threat. To ground this analysis, I draw on Foucault's genealogy of discipline and visibility, Morgan's account of modern suffering and Arnault's exploration of redemption. Emotions such as guilt, shame, compassion, elevation and hope are examined as morally significant emotions that can undermine or strengthen nurses' professional integrity. Evidence‐informed strategies are proposed for nursing leaders to transform moral distress into moral resilience through relational governance, psychologically safe practices and ethical policy design. The paper concludes that ethical clinical decision‐making is a whole‐body, socially embedded achievement and that cultivating ethical climates requires attending not only to rules and resources but also to how organizations shape the affective and relational environments in which nurses think, feel and act.

## 1 Introduction

I live daily with the reality that clinical judgement is never purely computational. A nurse at the bedside does not reason as a detached calculator; she senses, appraises and acts through a body attuned to suffering, risk and the social meanings that shape what is possible. When I consider the split‐second decisions that change outcomes such as recognizing opioid‐induced ventilatory impairment before it becomes catastrophic, noticing the subtle shift in a patient's breathing or deciding how firmly to push a reluctant team to revisit goals of care, I see the limbic brain quietly at work. The amygdala signals affiliation or threat; the hippocampus situates the present against a backdrop of prior events; and the ventromedial prefrontal cortex (vmPFC), anterior cingulate cortex (ACC) and insula integrate feeling and value into a sense of *what matters now* (A. Damasio 1994, 1999; Craig 2002). These systems mediate between the internal milieu (homoeostatic needs, visceral signals, autonomic states) and the external social environment of hierarchies, norms, policies and pressures that shape moral appraisal and action selection.

Within nursing ethics, ‘moral distress’ originally referred to knowing the right action yet being constrained from enacting it (Jameton 1984). Over time, this formulation has been revised as nurses rarely possess certain knowledge of the morally right action and instead, they form judgements or beliefs about what ought to be done within ambiguous and contested contexts (Fourie 2015). Moral distress often arises when these judgements meet institutional, interpersonal or structural barriers. Its consequences include emotional exhaustion, disengagement and compromised safety (Epstein and Hamric 2009). Neuroscience offers one important layer of understanding, showing how interoceptive prediction errors, threat detection and value judgements shape what clinicians notice and choose under pressure (Barrett 2017). Critical theory adds another layer: emotions and judgements are shaped within social worlds governed by visibility, discipline and power (Foucault 1995/1975). Institutions teach people what counts as risk, what counts as courage and what counts as defensible behaviour. They shape, reward and punish feelings.

My aims in this paper are threefold. First, I examine how the limbic brain integrates bodily and social signals into clinical decisions, grounding this analysis in contemporary affective neuroscience. Second, I critically assess Damasio's account of the relationship between emotion and reason, highlighting its enduring insights, conceptual limitations and implications for nursing. Third, I explore emotions of redemption, guilt, shame, compassion, elevation and hope, as pathways from moral distress towards moral repair, drawing on Arnault's philosophical work and empirical affective science. I write from the standpoint of my current role as a Director of Professional Practice, where I witness the cultural, relational and policy structures that either erode or enable ethical action. What follows is both an argument and an invitation to redesign clinical environments so that morally appropriate actions feel supported, hard truths become speakable and the limbic brain becomes an ally of ethical excellence.

## 2 The Limbic Brain as Mediator of Body and Social World

### 2.1 Interoception, Allostasis and Salience

Interoception refers to the brain's mapping of the body's internal state such as heart rate, respiration, visceral pressure, pain and numerous other autonomic cues. These sensations are not passive inputs; the brain predicts and interprets them through an allostatic control regime, anticipating bodily needs and preparing for action (Barrett 2017; Craig 2002). The insula and ACC play central roles in translating internal physiological conditions into felt states, while interacting with the amygdala and vmPFC to mark stimuli as salient or ignorable, threatening or safe, affiliating or alienating (A. Damasio 1999).

In clinical practice, these circuits underlie the nurse's felt responses long before explicit reasoning catches up. A subtle pallor, a rustle in a monitor, the uneasy sense that a medication order ‘doesn't look right’, these sensations may surface as limbic‐driven salience signals that orient attention and behaviour. These moments are not mystical intuition; moreover, they represent experience‐encoded, physiologically mediated predictions about risk and significance.

### 2.2 Social Context, Power and Moral Perception

Interoceptive cues do not arise in a vacuum; it is the brain that integrates internal signals with exteroceptive and social information to construct the ‘what matters now’. The same bradycardic trend on a monitor feels different in a calm, well‐staffed unit compared to that of an overstretched night shift already managing multiple deteriorations. A family's worried glance carries more weight when prior experiences of marginalization or distrust are known. The limbic brain is therefore not only a biological regulator but also a social organ, calibrated within contexts of hierarchy, surveillance, identity and institutional norms (Barrett 2017).

Foucault's genealogy of discipline and punishment helps reveal why these social conditions weigh so heavily on limbic appraisal. The modern clinic, like the prison or school, is built upon mechanisms of visibility, comparison and discipline that shape how people behave and how they feel (Foucault 1995, 1975). Dashboards, audits, performance reports and incident reviews are contemporary expressions of this logic.

When visibility shifts from learning to exposure, shame becomes a governing emotion. The amygdala's sensitivity to social threat is adaptive in such environments, as an example, if every escalation risks criticism, clinicians learn to keep their heads down; if every deviation risks humiliation, attention narrows to the measurable, even when the meaningful requires a broader view. These patterns are learned through repeated limbic memory interactions, with the hippocampus encoding the contexts in which speaking up led to reward or punishment, and the amygdala shaping the affective predictions that guide future encounters.

### 2.3 Memory, Learning and the Formation of Moral Habits

Memory circuits consolidate these social‐emotional patterns over time. When nurses are repeatedly dismissed or chastised for raising concerns, the amygdala influences hippocampal encoding such that threat‐related cues become tightly linked to certain contexts, people or actions. Conversely, when nurses are heard, supported and thanked, approach behaviours become more likely.

Organizations, much like individuals, develop affective habits. A unit may carry collective memories around certain clinical events, high‐risk physicians, particular procedures or patterns of leadership behaviour. These affective climates shape what feels possible. Because the limbic system mediates between body and world, the organizational world becomes part of the brain's operating environment. Governance, in this view, is not merely procedural; it is an affective architecture that shapes how people feel, respond and ultimately act within the system.

### 2.4 Predictive Processing, Chronic Threat and Normative Evaluation

To extend the discussion of how affect shapes moral perception, predictive processing offers an important account of the brain's broader regulatory dynamics. Rather than functioning as a passive recipient of interoceptive data, the brain actively generates predictions about bodily states and updates these predictions in relation to incoming sensory and interoceptive signals (Barrett 2017). Within this framework, feelings emerge from the dynamic interplay between actual physiological conditions and the brain's anticipatory models of those conditions.

Building on this account, predictive processing also clarifies why clinicians' affective perceptions may shift in environments marked by ongoing uncertainty or chronic threat. When institutional, interpersonal or structural pressures become persistent rather than episodic, the brain correspondingly recalibrates its predictive models. Empirical research demonstrates that sustained threat amplifies the precision of threat‐related predictions, thereby increasing the likelihood that ambiguous cues will be experienced as dangerous (Maddox et al. 2019; Zhang; Geng et al. 2021). Chronic stress likewise induces structural and functional sensitization of the amygdala, heightening its reactivity and promoting interpretations of neutral stimuli as potentially threatening (Zhang et al. 2018; Zhang, Zhang, et al. 2021; Blum 2024). In parallel, trauma‐exposed and chronically stressed populations show disruptions in interoceptive accuracy, with bodily sensations increasingly filtered through a threat‐oriented prior (Fani et al. 2024). These neurocognitive adaptations do not reflect personal weakness or individual error; rather, they are predictable consequences of sustained conditions of hierarchy, uncertainty and moral strain. This analysis provides an essential bridge to the ethical questions that follow, illustrating how the perceptual landscape within which clinicians form judgements is shaped, and at times constrained, by the environments in which they practice.

For the purposes of this argument, distortion is defined as a misalignment between interoceptive appraisal, the objective risk context and professional norms of practice. Chronic threat dynamics can shape appraisal in ways that lead clinicians towards silence, avoidance or defensive practice even when these responses undermine patient care. Clarifying the normative implications, therefore, requires addressing several issues directly. First, distortion is not determined by individual subjective experience. It is evaluated against shared professional norms, evidence‐based practice and collective ethical commitments, ensuring that appraisal remains situated within a relational and professional frame rather than idiosyncratic feeling. Second, compassion and moral elevation serve as counterbalancing forces. These self‐transcendent emotions, characterized by warmth, uplift and an expanded sense of connection, have been shown to broaden attentional scope and interrupt the narrowing effects of threat (Haidt 2003; Singer and Klimecki 2014). Their cultivation offers clinicians an additional resource for maintaining perceptual openness under conditions of strain. Finally, unsupported emotion poses the greater ethical risk. Emotions play a central role in moral perception, but unexamined or unsupported emotional responses can mislead. Moral psychology demonstrates that affective reactions vary across individuals and contexts and may generate divergent or biased judgements without reflective scrutiny (Landy and Kupfer 2023; Bucciarelli et al. 2023). As Nussbaum argues, emotions possess evaluative content but are not inherently reliable guides as they require critical reflection to distinguish morally insightful responses from those shaped by fear, bias or distorted appraisals (Nussbaum 2001). Clinical ethics scholarship similarly emphasizes that emotions become ethically informative only when situated within relational grounding, dialogue and structured moral reflection; otherwise, they risk becoming distortive rather than clarifying (Molewijk et al. 2011).

Clinical ethics scholarship similarly emphasizes that emotions become ethically informative only when situated within relational grounding, dialogue and structured moral reflection; otherwise, they risk becoming distortive rather than clarifying (Molewijk et al. 2011). Predictive‐processing accounts help explain *why* such distortions occur, as emotional appraisals can become biased under chronic stress or threat, reinforcing the need for normative ethical evaluation to guide *how* clinicians interpret and respond to these affective signals (Fani et al. 2024; Maddox et al. 2019).

## 3 Moral Distress, Emotions of Redemption, Clinical Decision Dynamics

### 3.1 Moral Distress

Moral distress is often recognized most clearly not through formal definitions but through lived clinical experience, for example, a nurse fighting back tears after discharging an older adult into an unsafe environment, or a charge nurse insisting on a safety pause despite subtle cues that her advocacy is unwelcome. These moments expose the affective and relational texture of moral conflict in practice. Jameton (1984) defined moral distress as knowing the right action yet being unable to carry it out. Contemporary scholarship has refined this framing, noting that clinicians rarely possess full epistemic certainty about what is morally required; instead, they form provisional judgements within ambiguous and contested contexts (Fourie 2015). Distress arises when internal or external constraints inhibit acting on those judgements (Epstein and Hamric 2009). This aligns with findings in moral psychology that emotions signal moral salience but cannot, on their own, determine moral correctness without reflection and evaluation (Bucciarelli et al. 2023).

From a neurocognitive perspective, episodes of moral distress can be understood as forms of limbic dysregulation under threat. Anticipated retaliation, shaming or futility heightens amygdala‐driven threat appraisal, consistent with research showing that stress amplifies fear circuitry and intensifies sensitivity to potential danger (Maddox et al. 2019). As perceived control diminishes, the stress‐response system mobilizes fight, flight or functional freeze responses. Clinically, this may manifest as hesitancy, voice tightening or behavioural inhibition that is misinterpreted as compliance. In some cases, the dorsal vagal complex may initiate shutdown responses that mimic calmness while reflecting autonomic overwhelm (Porges 2011).

In the aftermath of such events, clinicians may experience interoceptive prediction errors: rumination, intrusive replay, sleep disruption, narrowed attentional capacity and emotional exhaustion. Research on threat memory encoding demonstrates that the hippocampus and amygdala routinely rehearse morally charged moments, reinforcing somatic markers of shame, fear or self‐blame (Maddox et al. 2019). Simultaneously, the ACC continues to register unresolved value conflict, increasing cognitive burden and heightening vulnerability to future ethical missteps.

#### 3.1.1 Structural Contributors to Limbic Overload

Limbic dysregulation does not emerge in isolation; organizational and structural dynamics powerfully mediate clinicians' emotional and moral experiences. Moral psychology has demonstrated that moral emotions and judgements are deeply shaped by contextual, interpersonal and relational cues (Landy and Kupfer 2023). Performance dashboards and audits framed punitively activate shame‐based monitoring. Chronic understaffing and systemic scarcity normalize distress, generating organizational forms of learned helplessness. Hierarchical micro‐interactions such as a dismissive consultant, an impatient tone or memories of being ignored, reshape clinicians' predictions of social risk (Baez et al. 2017). Under such conditions, what ‘feels’ morally salient may reflect strategies for social survival rather than the actual ethical needs of the patient. In this sense, predictive processing becomes ethically consequential: the limbic system functions as designed, but in maladaptive environments, its predictions become misaligned with professional and moral standards.

#### 3.1.2 Consequences for Decision‐Making

Under threat, decision‐making narrows predictably. Heightened arousal constricts attentional scope, privileging immediate, defensible actions over more complex, patient‐centred responses (Landy and Kupfer 2023). Anticipatory shame inhibits upward communication, while moral disengagement may emerge as a coping mechanism, manifesting as depersonalization of patients or normalization of unsafe practices. These patterns reflect neurobiological adaptations to unsafe social conditions, not deficits of moral character. Crucially, because they are learned, they can be unlearned when conditions shift.

### 3.2 Emotions of Redemption: Guilt, Shame, Compassion, Elevation and Hope

If moral distress has a neurocognitive and relational trajectory, so too does redemption. Research on moral injury shows that morally injurious experiences can generate profound emotional disruption, but they may also initiate pathways of repair, meaning‐making and renewed moral agency (Williamson et al. 2021). Arnault (2003) describes redemption as a possibility rather than an inevitability, a refusal to allow suffering or moral failure to have the final word. This view aligns with emerging work on post‐traumatic growth, which demonstrates that individuals may move towards integration and restored agency when supported in reconstructing meaning, reaffirming values and re‐engaging relationally after moral disruption (Hoover and Metz 2024).

Recent research also highlights the role of self‐transcendent moral emotions such as compassion, elevation and hope in broadening attention, interrupting maladaptive threat responses and restoring ethical motivation (McGuire et al. 2025). Redemption, then, is both an emotional and a structural process: a re‐engagement with moral identity and collective values made possible through environments that cultivate reflection, connection and ethical dialogue (Williamson et al. 2021).

#### 3.2.1 Guilt and Shame

The distinction between guilt and shame is foundational in moral psychology and central to understanding clinicians' responses to morally distressing events. Guilt, ‘I did something wrong’, motivates reparative action, apology and accountability. It orients individuals towards the moral community and supports corrective behaviour. Whereas Shame, ‘I am wrong’, activates avoidance, concealment and defensiveness, narrowing attention and distorting moral judgement (Landy and Kupfer 2023). Shame‐based cultures silence communication, suppress learning and erode moral agency. In contrast, guilt‐supportive cultures clarify responsibility, affirm intrinsic worth and enable constructive moral repair. Research shows that the moral significance of these emotions lies not in the feelings themselves but in the appraisals that generate them (Baez et al. 2017). These appraisals determine whether emotions facilitate ethical engagement or contribute to moral paralysis.

#### 3.2.2 Compassion

Compassion and empathic distress, though often conflated, represent distinct affective states with different ethical implications. *Empathic distress* is self‐focused and depleting: ‘I cannot bear this’. It often results in avoidance or emotional withdrawal. *Compassion*, by contrast, is other‐focused and sustainable, grounded in a desire to alleviate suffering. Affective neuroscience demonstrates that compassion training increases prosocial behaviour, emotional regulation and resilience under stress (Singer and Klimecki 2014). Compassion functions as a neurobiological antidote to threat‐driven narrowing, broadening cognition and enabling clinicians to remain present in morally complex situations.

#### 3.2.3 Elevation

Elevation, described as feelings of warmth, uplift and expanded moral possibility, arises when witnessing acts of moral excellence (Haidt 2003). Elevation broadens attention, strengthens prosocial motivation and recalibrates limbic patterns towards approach rather than avoidance. This aligns with findings that moral emotions can dynamically reshape perception, appraisal and action (Landy and Kupfer 2023). Within clinical teams, stories of integrity or courage can shift the affective climate, counteracting cynicism and moral fatigue more effectively than procedural directives.

#### 3.2.4 Hope

Hope is not optimism; it is a form of cognitive openness under constraint, consistent with research showing that self‐transcendent moral emotions broaden perception and expand one's sense of possible action (Haidt 2003). Hope interrupts threat cascades, expands the perceived action space and supports the reflective and imaginative capacities needed for ethical problem‐solving. Moral psychology identifies hope as a forward‐looking moral emotion that sustains agency even in environments where structural barriers persist (Baez et al. 2017). Hopelessness collapses moral and cognitive horizons, whereas hope restores them.

#### 3.2.5 Redemption

Redemptive emotions guilt transformed into accountability, compassion emerging from distress, elevation sparked by witnessing moral excellence and hope sustained through uncertainty—are not inevitable outcomes. Their emergence depends on relational, cultural and organizational conditions. Clinical ethics scholarship emphasizes that reflective dialogue, relational grounding and shared deliberation are essential for transforming raw emotional reactions into moral insight (Molewijk et al. 2011).

Organizations, therefore, play a decisive role in shaping whether shame becomes paralysis or is transformed into repair, whether despair turns into possibility and whether moral distress becomes cumulative injury or a catalyst for renewed integrity. Redemption is both a personal and a structural accomplishment, an interplay between individual capacities and the environments that cultivate or constrain them.

### 3.3 Limbic–Moral Dynamics in Nursing Decisions: Case Examples

Clinical decision‐making in nursing is shaped not only by knowledge and technical skill but also by the dynamic interplay between limbic appraisal, moral judgement and organizational context. The following examples illustrate how neurobiological and cultural factors converge in moments of ethical significance.

Consider a postoperative patient receiving opioid analgesia. A bedside nurse notices increasing sedation and a slowing respiratory rate. Her physiological arousal intensifies signalled by heart rate rising and stomach tightening, as her attention narrows to the patient's chest movement. At the same time, she recalls that the attending physician has previously dismissed nursing concerns. In this moment, multiple neurocognitive systems are engaged as the amygdala evaluates both physiological and social threat; the vmPFC weighs competing values and anticipated outcomes; the ACC registers internal conflict; and somatic markers signal that ‘something is wrong’. Yet the predictive expectation of social dismissal or reprimand may override her patient‐centred impulse to escalate concerns. If she hesitates, the delay does not reflect incompetence but the neurobiological consequences of limbic conflict under hierarchical pressure.

A contrasting scenario demonstrates how organizational culture reshapes these appraisals. In a unit where escalation pathways are clear and psychologically safe, prior escalations have been met with gratitude rather than criticism, debriefings occur without humiliation and communication protocols provide standardized language for raising concerns, the nurse's somatic markers consolidate differently. The implicit prediction is not ‘escalation equals danger’, but ‘escalation equals safety and support’. Her limbic system interprets advocacy as socially reinforced rather than socially risky, enabling quicker, more confident action.

A second illustration involves an older adult who is medically stable but socially precarious at the time of discharge. The nurse anticipates risk and potential failure. Throughput pressures emphasize length of stay, and organizational metrics convey that efficiency is paramount. The ACC strains under competing obligations: the duty to ensure safe discharge and the systemic expectation to facilitate timely transitions. In an environment with strong interdisciplinary huddles and accessible community partners, the nurse's limbic appraisal shifts towards a sense of efficacy. Naming risk is not interpreted as futility but as an invitation to collaborative action.

These examples illustrate that clinically significant moral action is co‐produced by neurobiology and culture. Limbic responses shape what feels urgent or risky, while organizational structures determine whether those signals lead to advocacy, hesitation or disengagement. Ethical nursing practice thus relies not only on individual moral agency but on environments that align limbic predictions with professional standards and patient‐centred care.

### 3.4 Leadership and Practice Implications for Nursing

If emotions shape what feels possible within clinical work, leadership becomes the practice of intentionally shaping the affective architecture of healthcare environments. Leaders influence not only operational processes but also the limbic conditions under which clinicians perceive threat, experience psychological safety and enact moral judgement. Psychological safety research demonstrates that when interpersonal threat is reduced and clinicians feel free to speak up without fear of humiliation or retribution, decision‑making quality improves, communication becomes more transparent and team performance strengthens (A. C. Edmondson 2018; A. C. Edmondson and Lei 2014). Within nursing, this means that leaders must design systems in which morally appropriate actions are not merely cognitively understood but are also felt as feasible, supported and safe to pursue.

## 4 Ethically Aligned Clinical Policy

Clinical policies play a central role in shaping cognitive and emotional processes by providing predictable, ethically grounded pathways for action. When escalation routes are clear and consistently reinforced, clinicians experience reduced social threat because their advocacy is supported by organizational expectations and sanctioned processes (A. C. Edmondson 2023). Standardized clinical language similarly reduces cognitive load, strengthening clinicians' ability to articulate concerns under conditions of stress, a finding consistent with foundational work demonstrating that structured communication supports team learning and coordination (A. Edmondson 1999). Policies that integrate value‑based reminders, such as references to dignity, autonomy or equity, also help clinicians maintain moral considerations within their attentional field during high‑pressure situations. Together, these policy structures reduce uncertainty, reinforce ethical expectations and support decisive action when patient safety is at risk.

### 4.1 Psychological Safety and Just Culture

Psychological safety is cultivated through consistent relational behaviours rather than aspirational statements. A. C. Edmondson (2018, 2024) describes psychological safety as a shared belief that team members can voice concerns, raise questions and report errors without fear of embarrassment or punishment. Units characterized by high psychological safety show more robust learning behaviours, clearer communication patterns and fewer preventable adverse events. The principles of a Just Culture complement psychological safety by replacing individual blame with system‑level learning while retaining accountability for behavioural choices rather than unintended outcomes (Robichaux and Vittone 2023). Empirical studies show that Just Culture practices, such as open communication, reflective dialogue and non‑punitive error reporting, promote organizational learning and strengthen patient safety outcomes (Murray et al. 2023; van Baarle et al. 2022). These cultural conditions also diminish limbic responses to anticipated threat, freeing attentional and working‑memory resources for patient‑centred reasoning and re‑associating the act of speaking up with support rather than sanction.

### 4.2 Training for Interoceptive and Affective Skills

Professional development in nursing must address affective and interoceptive competencies alongside traditional clinical skills. Emotional and physiological regulation is essential for clinicians practicing in settings characterized by uncertainty, acuity and moral complexity. Practices such as paced breathing and brief body‑scanning exercises enhance interoceptive accuracy and autonomic regulation. Compassion cultivation further strengthens clinicians' capacity to remain present and engaged during morally challenging interactions; neuroscientific evidence shows that compassion training increases prosocial behaviour, emotion regulation and resilience under stress (Singer and Klimecki 2014). When combined with cognitive reappraisal and values‑based action training, these practices help clinicians link moment‑to‑moment decisions with their professional identity and ethical commitments. Importantly, such training must be embedded at the team and organizational levels to avoid placing the burden of regulating systemic stress solely on individuals.

### 4.3 Timely Ethics Support

Ethics support must be integrated into the everyday rhythms of clinical practice rather than isolated to extreme or catastrophic cases. Low‑barrier ethics consultation pathways, routine moral distress rounds and interdisciplinary huddles help identify emerging ethical tensions early and facilitate collective interpretation. These structures are essential because moral injury is exacerbated when clinicians feel isolated, unable to voice their concerns or uncertain about the normative dimensions of their distress (Williamson et al. 2021). Routine ethics engagement normalizes reflection, strengthens relational trust and fosters a shared sense of responsibility for ethical decision‑making across the team.

### 4.4 Governance, Metrics and Redeemed Visibility

Although visibility in healthcare can function as a disciplinary technology, it can also foster ethical clarity and collective morale when oriented towards recognition and learning rather than surveillance. Metrics and dashboards that reward high‑quality escalation, thoughtful communication, moral courage and effective interdisciplinary collaboration counteract punitive audit cultures and reinforce desired ethical behaviour. Storytelling, by public acknowledgement of near misses avoided, advocacy enacted or meaningful patient relationships forged, elicits moral elevation, a self‑transcendent emotion shown to broaden moral imagination and enhance prosocial motivation (Haidt 2003; McGuire et al. 2025). When used intentionally, governance and evaluation processes promote ethical climates that support rather than undermine clinicians' moral commitments.

### 4.5 Anticipating Moral Friction in Policy Design

Effective clinical policy anticipates predictable moral tensions and provides structured, psychologically safe pathways for navigating them. For example, policies addressing care for patients who use substances must explicitly acknowledge structural vulnerability to prevent cycles of stigmatization, fear and avoidance. Similarly, policies guiding disagreements in treatment planning should provide tiered and time‑bound escalation procedures. When disagreement is normalized and structured within safe processes, its limbic meaning shifts: speaking up becomes conceptualized as a valued contribution to care rather than a socially risky or oppositional act.

### 4.6 Reframing Damasio for Nursing Leadership

Damasio's work suggests that emotion and reason are inseparable in practical judgement, where bodily signals shape cognition, attention and ethical decision‐making (A. R. Damasio 1998). Nursing leadership must therefore cultivate environments in which somatic markers orient clinicians towards clarity, courage and patient‑centeredness. This includes reducing chronic threat, supporting compassion‑based motivation, ensuring procedural clarity, cultivating relational trust, rewarding moral courage and using performance metrics to promote learning rather than fear. Both psychological safety research and Just Culture scholarship highlight the need for governance tools, such as visibility, monitoring and comparison, to be used intentionally and judiciously to avoid replicating punitive environments (A. C. Edmondson 2018; Robichaux and Vittone 2023). Redemptive accountability balances rigour with compassion by addressing harm while supporting practitioners who navigate complex clinical systems. In such systems and environments, emotion and reason collaborate rather than compete.

### 4.7 Integrating Suffering and Redemption in Clinical Culture

Healthcare exists in a paradox: it aims to alleviate suffering even as systemic demands, resource constraints and defensive practices can inadvertently reproduce it. Morgan (2002) characterizes modern organizations as producing bureaucratized forms of suffering, while Arnault (2003) frames redemption as the countervailing possibility grounded in repair, restoration, reform and remembrance. In clinical practice, redemption manifests through sincere apology and corrective action after harm, attention to clinician well‑being in the aftermath of moral injury, structural reform that addresses the root causes of moral distress and acts of remembrance that honour patients and transform grief into renewed commitment. Over time, these practices recalibrate the limbic system: truth‑telling strengthens connection, compassion enhances efficacy and courage builds community. Through this embodied and relational learning, clinical cultures are remade into environments where ethical practice becomes not an act of resistance but the expected, supported standard of care.

## 5 Conclusion

Clinical decision‐making in nursing is an embodied, relational and socially situated process. The limbic system plays a central role in mediating between interoceptive signals and the external social world, generating the felt sense of salience that shapes what clinicians notice, prioritize and act upon. Damasio's work restores emotion to its rightful place in practical reasoning by demonstrating that somatic and affective cues are integral, and not peripheral, to judgement. Contemporary neuroscience and social theory extend this insight by showing that emotional signals are predictive, plastic and profoundly conditioned by institutional environments.

Moral distress emerges when there is a disruption in the alignment between physiological salience, professional judgement and the social feasibility of ethical action. In environments characterized by chronic threat such as in hierarchical suppression, punitive responses or sustained resource scarcity, limbic appraisals become distorted. Salience narrows (risk feels amplified), and the cognitive and affective groundwork for moral agency becomes compromised. Such distortions heighten the risk of moral injury by undermining clinicians' capacity to act in accordance with their values.

Redemption offers a countervailing path by restoring alignment through both emotional and structural shifts. When clinicians move towards guilt rather than shame, compassion rather than empathic distress, elevation rather than cynicism and hope rather than despair, coupled with an organization provides the supports needed for ethical action, moral orientation can be recalibrated. In this sense, redemption is not solely an individual transformation but also an organizational achievement.

For nursing leaders, the imperative is clear: create ethical climates that align somatic markers with professional values. This requires psychologically safe governance, redemptive accountability, relationally attuned practice design and policies that anticipate rather than ignore moral friction. When organizations reliably support courageous care, clinicians' emotional and cognitive systems work in concert rather than conflict. In such environments, the limbic brain becomes an ally of ethical excellence. When ethical culture, leadership practice and neurobiological realities converge, the everyday decisions of nurses accumulate into clinical environments where patients, families and healthcare workers can flourish.

## Funding

The authors have nothing to report.

## Ethics Statement

This manuscript is a scholarly and theoretical analysis, drawing on published literature, professional experience and ethical reflection. It does not report empirical research, involve human participants or include the collection or analysis of identifiable data. As such, research ethics board review was not required. The analysis is informed by sustained engagement with clinical practice and leadership context, but all examples and reflections are presented at a level of abstraction that preserves confidentiality and avoids references to specific individuals or cases.

## Conflicts of Interest

The author declares no conflicts of interest.

## Data Availability

No new data was generated or analysed in support of this research. All sources cited are publicly available.
